# Computational study of noise in a large signal transduction network

**DOI:** 10.1186/1471-2105-12-252

**Published:** 2011-06-21

**Authors:** Jukka Intosalmi, Tiina Manninen, Keijo Ruohonen, Marja-Leena Linne

**Affiliations:** 1 Department of Mathematics, Tampere University of Technology, P.O. Box 553, 33101 Tampere, Finland; 2 Department of Signal Processing, Tampere University of Technology, P.O. Box 553, 33101 Tampere, Finland

## Abstract

**Background:**

Biochemical systems are inherently noisy due to the discrete reaction events that occur in a random manner. Although noise is often perceived as a disturbing factor, the system might actually benefit from it. In order to understand the role of noise better, its quality must be studied in a quantitative manner. Computational analysis and modeling play an essential role in this demanding endeavor.

**Results:**

We implemented a large nonlinear signal transduction network combining protein kinase C, mitogen-activated protein kinase, phospholipase A2, and *β *isoform of phospholipase C networks. We simulated the network in 300 different cellular volumes using the exact Gillespie stochastic simulation algorithm and analyzed the results in both the time and frequency domain. In order to perform simulations in a reasonable time, we used modern parallel computing techniques. The analysis revealed that time and frequency domain characteristics depend on the system volume. The simulation results also indicated that there are several kinds of noise processes in the network, all of them representing different kinds of low-frequency fluctuations. In the simulations, the power of noise decreased on all frequencies when the system volume was increased.

**Conclusions:**

We concluded that basic frequency domain techniques can be applied to the analysis of simulation results produced by the Gillespie stochastic simulation algorithm. This approach is suited not only to the study of fluctuations but also to the study of pure noise processes. Noise seems to have an important role in biochemical systems and its properties can be numerically studied by simulating the reacting system in different cellular volumes. Parallel computing techniques make it possible to run massive simulations in hundreds of volumes and, as a result, accurate statistics can be obtained from computational studies.

## Background

Noise plays an essential role in nearly all biochemical systems. It derives from reaction events that are discrete and occur at random times. The structure of a particular biochemical reaction network (BRN) determines the way the system evolves and defines the quality of noise accordingly. Consequently, there exist several types of noise processes which occur in these systems. Noise induced effects can have both a quantitative and qualitative impact on the behavior of a biochemical system [1]. Knowing the characteristics of noise processes would help develop better models and understand the underlying principles of the biological phenomena better.

The effects of noise in biochemical systems have been studied to some extent but the understanding of noise is still rather deficient. A common misconception is that noise is always a disturbing factor in a biological system. Contrary to this popular belief, noise might in some cases be the factor which keeps the system functioning properly [2]. Noise can, for example, make the system more robust to external perturbations or it might lead to a specific behavior like noise-induced bistability with oscillations [3]. The importance of noise has been emphasized especially when the focus has been on the signaling networks related to memory and learning or gene regulatory networks (see e.g. [4-6]). The frequency content of noise and its relation to the structure of gene regulatory networks have also been studied recently [7-9]. Computational methods and models are outstandingly useful when stochastic effects and noise in BRNs are studied. There exist several stochastic modeling formalisms that enable the time-evolution of BRNs to be studied in theory and using computer simulations [10]. In the field of computational systems biology, the exact Gillespie stochastic simulation algorithm (SSA) [11,12] and its several variants are the most commonly used stochastic simulation procedures. The drawback of the Gillespie SSA is the computational burden that increases when the number of interacting chemical particles in the system increases. Several approximative simulation approaches have been developed to decrease computing times (see e.g. [13-15]). The conditions in which the approximations are valid are, however, often hard to specify and this makes the selection of the simulation method a demanding task. The computing times of exact simulation procedures, such as the Gillespie SSA, can also be decreased by applying parallel computing (see e.g. [16]). This approach is especially attractive if, for example, the statistical characteristics of a biochemical process need to be estimated via simulation. In addition to simulation procedures, there exist also non-simulative approaches which can be used, for example, to numerically estimate noise levels (see e.g. [17]).

Besides the stochastic modeling and simulation of BRNs, computational methods are invaluable in the analysis of biochemical data. The data, obtained from time-series simulations or from laboratory experiments, can be numerically studied both in the time and frequency domain. Out of these two, the time domain analysis is the traditional approach. Typical time domain statistics are the mean, variance, autocorrelation, etc. which can be used to characterize the behavior of time-series. Frequency domain analysis, often used by engineers and physicists, provides other kind of information about the system. Using the frequency domain approach it is possible to decompose a biochemical signal into its frequency components and to study the magnitude of fluctuations at different frequencies. Fluctuations, both random and deterministic, are important in the functioning of biological systems. Even simple BRNs can be selectively responsive to specific frequency ranges [18,19]. The importance of periodic changes in chemical concentrations being widely known, it is surprising to notice that most simulation studies do not provide even a rough survey of frequency domain behavior. Some studies present analytical results for the signal processing properties of BRNs (see e.g. [18,19]) and the frequency domain characterization of biochemical noise (see e.g. [8,9,20]). These approaches, however, are often suitable only for linear or small networks, require an unbearable amount of calculations, or have other restrictions.

In this study, we utilize a straightforward numerical approach to explore noise in a biologically realistic BRN using simulated data. We implement a large nonlinear signal transduction network combining protein kinase C (PKC), mitogen-activated protein kinase (MAPK), phospholipase A2 (PLA2), and *β *isoform of phospholipase C (PLC*β*) networks. This BRN consists of 66 chemical species and 110 one-way reactions. In general, stochastic simulation of large networks of this kind is challenging and thus several technical aspects have had to be taken into account when implementing the network. The network is originally published by Bhalla and Iyengar in 1999 [21] and its parts have been studied to some extent using both deterministic and stochastic modeling methods [15,21-26]. In this study, we perform massive Monte Carlo simulations for the large network by applying parallel computing. As a stochastic simulation method we use the exact Gillespie SSA. We run simulations altogether in 300 different cellular volumes and compute the time and frequency domain characteristics of the noise processes for each volume. This kind of approach provides us with an overall picture of the noise in the system as a function of system volume. We show how basic frequency domain methods can be applied and what advantages they have compared to the time domain methods.

## Methods

### Stochastic modeling of BRNs

BRNs can be modeled using numerous different formalisms. To the modeler, a biochemical system can be perceived as a container full of particles that have certain sizes and velocities. When these particles (chemical species) collide, they react with some probability and produce other species [27]. The well-established theory of molecular dynamics describes how these chemical reactions occur at the molecular level and, in principle, we are capable of describing the dynamics of reacting species in detail [27]. In real systems, however, the amount of particles is large and it is impossible to track each and every molecule. Based on the theory of stochastic chemical kinetics, these systems can often be assumed to be well-stirred. This means that the particles are uniformly distributed over the volume and, in order to understand the time-evolution of the system, we need to keep track only of the numbers of particles of each species [27]. Gillespie has done pioneering work in describing the time-evolution of a well-stirred chemical system in terms of continuous-time discrete-state Markov processes [11,12]. He has also developed the formalism which enables us to simulate the Markov model as a straightforward computer algorithm, nowadays known as the Gillespie SSA. By means of the simulation algorithm we are able to generate realizations of the underlying stochastic process. A sufficient number of independent realizations can then be used to compute accurate statistical characteristics describing the process [28]. In most of the cases, it is impossible to obtain these characteristics analytically and, thus, simulation algorithms like the Gillespie SSA provide us with invaluable tools. In the following, we briefly recapitulate the theory behind the continuous-time discrete-state Markov model and Gillespie SSA.

Stochastic processes having the so-called Markov property (i.e. Markov processes) are by far the most important in physics and chemistry [29]. The Markov property states that the future behavior of the process depends only on the current state of the system. In the context of biochemical systems, this assumption can often (but not always) be accepted and Markov processes provide us with a well-established modeling formalism. In order to construct a Markov model for a biochemical system, we need to introduce some terminology. Let us consider a biochemical system consisting of *n *chemical species *X_i_*, *i *= 1, ..., *n*, and *m *reactions R*_j_*, *j *= 1, ..., *m*, and let **X**(*t*) be a stochastic process describing the time-evolution of the system. Each reaction R*_j _*in the system can be characterized by a propensity function *a_j_*(**X**) so that *a_j_*(**X**)Δ*t *gives the probability that the reaction R*_j _*will occur during the finite time interval Δ*t *[11]. The propensity functions depend only on the current state of the system and thus the Markov property is satisfied. With each reaction event we associate the so-called stoichiometric vector **v***_j_*, so that when the *j*th reaction occurs, the state of the system is updated by **X **+ **v***_j_*. In addition, we assume that the initial state of the system **X**(*t*), *t *= 0 is known.

Using the notation above, the system can be fully characterized by a continuous-time discrete-state Markov process. By denoting the probability that the system is in the state **x **at time *t *given the system is in the state **x**_0 _at time *t*_0 _by *p*(**x**, *t|***x**_0_, *t*_0_) and assuming that only one reaction can occur during *dt*, we can write(1)

Consequently, the time evolution of the probabilities can be described by a set of coupled differential equations which can be written in the form(2)

where *p*(**x**, *t|***x**_0_, *t*_0_), *a_j_*, and **v***_j _*are as described above [27]. This equation is called the chemical master equation (CME).

Based on the formalism described above, we can construct the CME for any BRN of interest. The problem is that the CME is analytically and even numerically intractable. Although the solution of CME can in some cases be solved or approximated (using e.g. the finite state projection algorithm [30]), the numerical simulation of the underlying Markov process is often practical. The Gillespie SSA [11,12] is the most popular procedure for this purpose. It can be derived from the CME without additional assumptions or approximations and is exact in that sense. A detailed derivation of the Gillespie SSA can be found in the references [11,12,27].

The Gillespie SSA has proven to be useful in several biochemical simulation studies, ranging from studies of gene expression to stochastic ion channel dynamics (see e.g. [31,32]). In some cases, however, the algorithm becomes computationally heavy. This is the case especially if the size of the system is large (i.e. the number of particles in the system is large) and reactions occur more frequently. In such situations, we have to consider approximations which are usually based on the time-discretization of the continuous-time process [27]. In this study, we simulate a reaction network in which the numbers of molecules in some chemical species are relatively small and thus the approximations are not valid. Consequently, our simulations are carried out using the exact Gillespie SSA. To be able to run massive simulations in a reasonable time, we apply parallel computing techniques.

### Deterministic modeling of BRNs

In the previous, we have described how biochemical systems containing only small numbers of molecules can be modeled in detail. Sometimes, however, random effects may be neglected and simpler, deterministic models can be used. When large chemical systems which contain huge numbers of molecules are concerned, random fluctuations tend to average out and the time-evolution of the system can be modeled using a continuous-time continuous-state deterministic ordinary differential equation (ODE) model. The traditional ODE model is based on the law of mass action, and like Gillespie [27] has shown, the model is asymptotically equivalent to the stochastic model when the volume of system is increased. According to the law of mass action the dynamics of a chemically reacting system can be described by the equation(3)

where **S **is the stoichiometric matrix containing the stoichiometric vectors as columns and the state-dependent vector **u **describes the reaction rates. In this study, the deterministic ODE model is used to determine the deterministic steady-state of the system.

### Analyzing simulation results in time domain

Simulation results are traditionally analyzed in the time-domain by computing the time-dependent sample mean and sample standard deviation of a stochastic process (see e.g. [15]). These characteristics are indeed useful as we are dealing with stochastic processes related to biochemical applications. Another useful statistical number is the coefficient of variation which we use in this work. The coefficient of variation can be computed using the formula(4)

where *σ*(*t*) is the (sample) standard deviation of the process and *μ*(*t*) is the sample mean. If the stochastic process is stationary (i.e. its statistical properties do not change in time), we can leave the time variable *t *out. The coefficient of variation provides us with the information of how strong the noise is compared to the mean level of the signal. In addition to the characteristics mentioned above it is sometimes useful to study the distribution of the process. Similar to the estimates for the mean, variance, and coefficient of variation, the distribution of the process can also be approximated using a large number of independent realizations. The approximated distribution can then be illustrated for example using histograms.

### Analyzing simulation results in frequency domain

Although the time domain analysis often provides important information about the biochemical system of interest, it still gives quite a limited insight into the (often non-linear) system. A broader view can be obtained by combining the time domain analysis with the frequency domain analysis. This approach provides us with information about the fluctuations typical for the particular system and the quality of noise arising from molecular interactions in general. In the following, we discuss frequency domain analysis, define terminology, and present a straightforward way of obtaining a rough approximation of the frequency domain behavior of a biochemical system through numerical frequency domain analysis.

**Figure 1:**
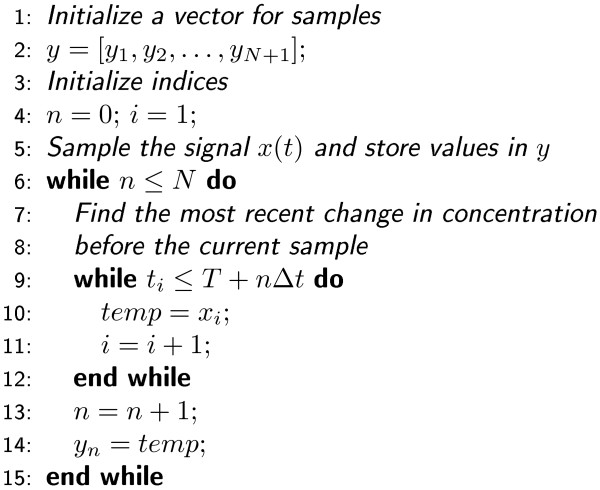
**Pseudocode for sampling algorithm**. A biochemical signal *x*(*t*) obtained from Gillespie SSA simulation can be characterized using two vectors **x **= [*x*_1_, *x*_2_, ..., *x_M _*] and **t **= [*t*_1_, *t*_2_, ..., *t_M _*]. Time points *t_i_*, *i *= 1, 2, ..., *M*, define when the concentration has changed and *x_i_*, *i *= 1, 2, ..., *M*, describe the concentration within the interval [*t_i_*, *t_i_*+1), *i *= 1, 2, ..., *M *-1. These vectors, as well as the starting time for sampling (*T*), the number of samples (*N*), and the time step (Δ*t*), are given as parameters for the algorithm.

**Figure 2:**
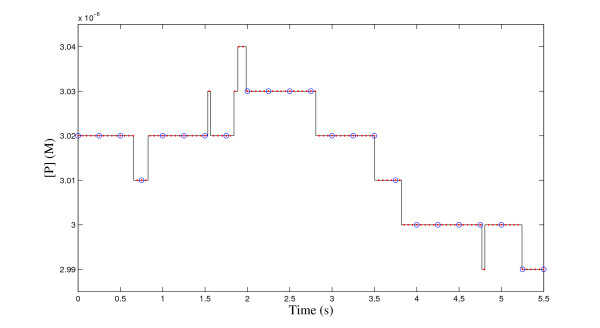
**Operation of sampling algorithm**. The continuous-time signal representing the concentration of chemical species P is sampled using the time step of 0.05 s (red dots) and of 0.25 s (blue circles). The small time step detects all details of the signal whereas the large time step neglects rapid changes.

The starting point for numerical frequency domain analysis is sampling. This means that a continuous-time signal *c*(*t*), ∞ ≤ *t *≤ ∞, is sampled at discrete time points *n*Δ*t*, *n *= 0, ± 1, ± 2, ..., and, as a result, we have a discrete signal *c*[*n*]. The choice of the time step Δ*t *(*s*) determines how frequently the signal is sampled. The reciprocal of the time step is called the sampling frequency (denoted by *F*) and, in order to capture all details of the original signal, it should be twice as much as the fastest oscillation in the signal [33]. However, this requirement is difficult to fulfill when realizations generated by the Gillespie SSA are analyzed. The realizations often contain rapid changes and thus the sampling frequency would have to be unreasonably high. Based on our numerical tests, however, the fastest changes usually do not seem to have a lot of power and, as an approximation, we can content ourselves with a lower sampling frequency. Pseudocode for our sampling algorithm is given in Figure 1 and the operation of the algorithm is exemplified in Figure 2. The algorithm enables us to sample the signal so that the most rapid changes are neglected.

After sampling, the signal is often down-sampled by some factor in order to adjust the frequency range of the frequency domain representation to the desired level. By altering the sampling frequency and the length of the time window (which is used to define a finite sequence from the infinite signal), it is possible to extract different kind of information from the signal. As biochemical systems often operate on various time scales, it is natural to pay attention to the selection of frequency range. If low frequencies are of interest, down-sampling of the signal is required. Before down-sampling, the signal must be low-pass filtered. Otherwise the high frequency fluctuations will aliase on the other frequencies in the frequency domain representation (for details see e.g. [33]). The filtering can be carried out using any available low-pass filter. The MATLAB^® ^function 'decimate' practically combines filtering and down-sampling and we highly recommend it.

The most essential part of the frequency domain analysis is the estimation of the actual frequency content of the signal. The frequency content can be estimated using a wide variety of methods. The methods include, for example, the standard periodogram method, Blackman-Tukey method, autoregressive model, maximum likelihood method, etc. (for a review, see [34]). In this study, we use the standard periodogram method as it is straightforward to implement and use. The standard periodogram approach can also be easily modified to fit for different kind of systems and it gives a good overall picture of the frequency domain behavior. If a more detailed time-frequency representation of a chemical system is of interest, one should use more advanced, non-stationary data processing methods (for a review, see e.g. [35]). Although these methods are more complicated to implement and use, they are in some cases required. The standard periodogram method is well-applicable if we are dealing with a process which is approximately stationary (at least within a suitably short time window).

The standard periodogram method is based on the discrete Fourier transform (DFT). The DFT for a finite discrete signal *c*[*j*], *j *= 0, ..., *N - *1, is mathematically defined by(5)

In practice, the DFT is computed using the fast Fourier transform (FFT) algorithm. A weighting window (e.g. Blackman window, Hamming window) is often applied to the signal to be transformed before computing the DFT to prevent the bias caused by the finite length of the signal [36]. The actual (one-sided) power spectral density (PSD) estimates can then be computed using the equation(6)

where we assume that *N *is always chosen to be even and each *f_k _*= *Fk*/*N *, *k *= 1, ..., *N*/2, presents a positive frequency [37].

### Parallel computing

The basic idea of parallel computing is to divide a computationally intensive routine into independent subtasks and execute them in parallel on multiple processors [38]. When computationally heavy Monte Carlo procedures, such as the Gillespie SSA, are used, carefully implemented parallelism can be used to reduce computing times significantly. In this study, we simulate the large network in 300 different cellular volumes. The serial execution of these simulations would be in practice impossible but the parallel simulation of all volumes can be carried out in a few days. We implement parallelism using the parallel computing platform (PC Grid) provided by Techila Technologies Ltd.

## Results

### Simulation setup

**Figure 3:**
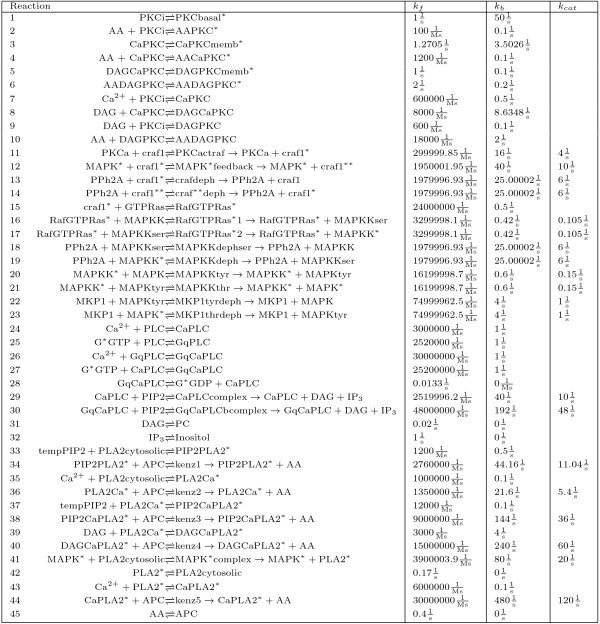
**Biochemical reaction network combining PKC, MAPK, PLA2, and PLC***β ***networks**. Reaction network combining PKC, MAPK, PLA2, and PLC*β *networks. The forward, backward, and catalyzing reaction constants are given by *k_f _*, *k_b_*, and *k_cat_*, respectively. The species APC, tempPIP2, Inositol, PC, and PIP2 are treated as constant model inputs and have the concentrations 30 × 10*^-^*^6 ^M, 2.5 × 10^-6 ^M, 0 M, 0 M, and 2.5 × 10*^-^*^6 ^M, respectively. The other 61 species are treated as model variables.

In this study, we simulated a large nonlinear signal transduction network that combines the PKC, MAPK, PLA2, and PLC*β *networks and analyzed the simulation results in both the time and frequency domain. The reactions of the network are presented in Figure 3. From now on, we refer to this network as the large network. We implemented all simulation procedures as well as analyzed the results in MATLAB^®^. In order to run the simulations in a reasonable time, we used the parallel computing platform (PC Grid) provided by Techila Technologies Ltd.

In the simulations, we first used the ODE model to determine the deterministic steady-state of the system and then simulated the actual noise processes around the steady-state in 300 different cellular volumes. As a stochastic simulation algorithm we used the Gillespie SSA. The simulated volumes were equidistantly spaced between 5 × 10*^-^*^16 ^l (comparable, for example, to the volume of the dentritic spine) and 10*^-^*^13 ^l (comparable, for example, to the volume of a cell). We sampled the simulated noise processes using the sampling algorithm presented in Figure 1. The sampling frequencies were 10^3 ^- 10^5 ^Hz. They were chosen depending on the properties of the sampled signal so that at least 95 percent of the power of the original signal was captured. The resulting signals were then filtered and down-sampled to obtain the desired frequency range. In the estimation of PSDs, we simply used a rectangular window. The PSDs were smoothed by summing adjacent frequency bins.

### Time domain characteristics of noise

**Figure 4:**
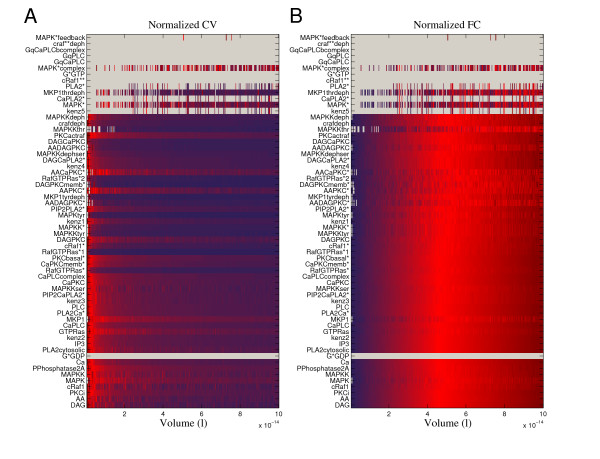
**Normalized coefficients of variation and normalized frequencies of change**. (A) Normalized coefficient of variation (CV) and (B) normalized frequency of change (FC) for all model species in different volumes are illustrated by color coding (heat map). The CV describes the strength of noise and FC tells how many times the concentration changes on average in a given time period. The values for CV and FC were estimated using 100 second signals (simulated noise processes). The simulated volumes were equidistantly spaced between 5 × 10*^-^*^16 ^l (comparable, for example, to the volume of the dentritic spine) and 10*^-^*^13 ^l (comparable, for example, to the volume of a cell). The rows in the heat maps are sorted in an ascending order according to the initial concentrations of species. The CV and FC values are normalized by their maximum values. The intensities in the heat maps vary from values close to zero (dark blue) to 1 (dark red). The zero CV and FC values are mapped to gray points. The large network includes species that stay at zero concentration in all studied volumes (craf**deph, GqCaPLCbcomplex, GqPLC, GqCaPLC, G*GTP, cRaf1**) as well as a species remaining at a constant concentration (G*GDP). As supposed, the strength of noise seems to decrease and the frequency of change increase when the volume is increased.

Biochemical information processing occurs in various cell organelles, all of them having different volumes. In order to learn how noise changes as a function of volume, we simulated the large network in different volumes. The network includes 61 non-input chemical species and in order to get an overall understanding of the behavior of these species, we computed the coefficients of variation and the frequencies of change for each species in each volume. The frequency of change (FC) is defined to be the number of changes in the molecular concentration within a certain time period. These characteristics were estimated using 100 second realizations and they are computed over time. The results are shown in Figure 4. The gray areas in the heat maps indicate that certain species maintain either a zero concentration (craf**deph, GqCaPLCbcomplex, GqPLC, GqCaPLC, G*GTP, cRaf1**) or a constant concentration (G*GDP) in all studied volumes. The non-active role of G*α*GDP (G*GDP, G*α *is a subunit of G protein, GDP is guanosine diphosphate) can be explained by the fact that the concentration of the activated form of enzyme PLC*β *(GqCaPLC, PLC*β *is the *β *isoform of phospholipase C) remains at zero concentration in all simulated volumes. This and other non-active species seem a bit suspicious from the biological point of view but, on the other hand, we have to keep in mind that this network is a subnetwork that has been extracted from a larger network and thus some parts of the network do not necessarily produce the natural level of activity. The rows (species) in Figure 4 are sorted in an ascending order according to the initial concentrations of species. It is important to notice that in the smallest volume 39 species have a zero concentration as an initial value. In the largest volume, the number of initial concentrations equal to zero is 18. The most of the species however seem to be active in all volumes even though the initial concentration was zero. When the molecular concentration is low, the discrete nature of chemical reactions is emphasized and consequently also the noise level is fairly high when compared to the average signal level. This can also be concluded from the coefficients of variation in small volumes (Figure 4A).

To pick up an interesting species producing irregular behavior, we consider the active form of mitogen-activated protein kinase (MAPK*) and discuss its behavior in small and intermediate volumes. In these volumes, the MAPK* concentration is very low or zero, as we can see in Figure 4. There are simulation runs during which the concentration stays at zero and, on the other hand, there are irregularly occurring realizations showing activity. The MAPK* thus represents some kind of non-stationary behavior and it would be interesting to see how its behavior reacts to different kinds of external stimuli in different volumes. We however leave this for a future work and do not try to make any biological conclusions based on the current results.

In general, the strength of noise seems to decrease and the frequency of change increase when the volume increases. This of course fits well to the theory of stochastic chemical kinetics. When the numbers of molecules increase in the system, the reactions occur at a higher rate and stochastic effects tend to average out. Most of the noise processes in the model behave relatively well and the noise is attenuated when the volume increases. It is however important to note that the strength of noise changes in a different manner for different species. For example, the noise strength in inositol trisphosphate (IP3) seems to decrease much faster compared to the strength of noise in protein phosphatase 2A (PPhosphatase2A) concentration (Figure 4A). Similar differences can also be observed between different species when comparing how FC changes as a function of volume (Figure 4B).

The molecules that are present in low numbers seem to produce the most irregular and unpredictable behavior. Heavy stochastic fluctuations can however be observed also in species that are present in higher concentrations. For example, arachidonic acid (AA) and diacylglycerol (DAG) have high concentrations compared to the other model species but they still produce notable fluctuations especially in small volumes. In the reaction network, AA and DAG are linked to the species that are present in low concentrations and it is likely that the heavy fluctuations in small volumes are due to these interactions. To track down the source of these fluctuations is another interesting question that we will leave for further studies.

Although we have observed irregular behavior and heavy fluctuations besides nicely behaving noise processes in the network, it seems that none of the model species deviates far away from the deterministic steady-state. This seems to tell something about the robustness of the network, although we did not perform inclusive analysis of the model dynamics (e.g. bifurcation analysis). Noise seems, according to our analysis, to have more impact on the behavior of the system when the system volume is small. The coefficients of variation computed in different volumes show that the random fluctuations are notably stronger in small volumes than in larger volumes. Therefore, the use of stochastic modeling and simulation methods is especially important if we are modeling biochemical systems in small volumes.

### Estimating frequency domain behavior

**Figure 5:**
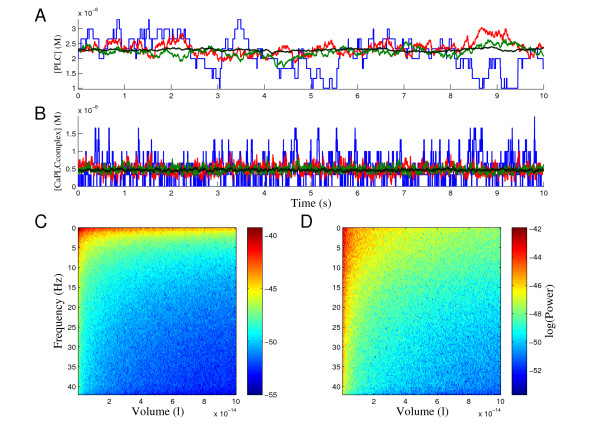
**Behavior of PLC and CaPLCcomplex, realizations and estimated PSDs**. (A) Realizations for PLC. (B) Realizations for CaPLCcomplex. The realizations are simulated using the Gillespie SSA in four different system volumes, 5 × 10*^-^*^16 ^l (blue), 3.5 × 10^-15 ^l (red), 10*^-^*^14 ^l (green), and 10^-13 ^l (black). The discrete nature of reactions can be easily seen in the smallest volume. In larger volumes, the strength of noise is smaller but still detectable. (C) Frequency domain behavior for PLC. (D) Frequency domain behavior for CaPLCcomplex. The estimated PSDs for PLC and CaPLCcomplex in different volumes (5 × 10^-16 ^- 10^-13 ^l) are illustrated by representing the frequency content of signals by color coding (heat map). The PSDs were estimated using 10 second signals (simulated noise processes). The noise in both species has more power on lower frequencies than higher frequencies.

**Figure 6:**
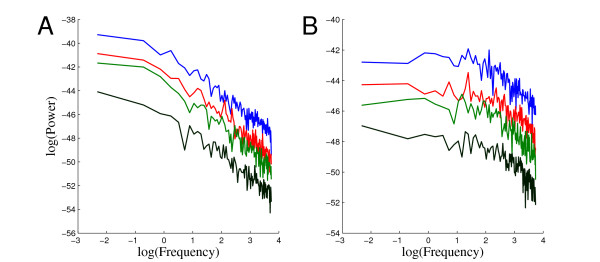
**PSDs in log-log-scale for PLC and CaPLCcomplex**. The estimated PSDs in the log-log-scale for PLC and CaPLCcomplex in four volumes. (A) PSDs for PLC. (B) PSDs for CaPLCcomplex. The color coding of the PSDs as well as the volumes used are the same as in Figure 5 (A and B).

The dependence between the system volume and the quality of noise can also be studied in the frequency domain. In general, the frequency domain analysis of the simulation results shows that most of the power in these noise processes is on the low frequencies. The quality of the PSD estimates depends somewhat on the stationarity of the noise processes studied. In order to illustrate the volume dependence of the quality of noise in the frequency domain, we have selected two model species: phospholipase C (PLC), and calcium phospholipase C complex (CaPLCcomplex). Illustrative realizations of these species are shown in (Figure 5A and 5B). The realizations are simulated in four different system volumes, 5 × 10^-16 ^l (blue), 3.5 × 10^-15 ^l (red), 10^-14 ^l (green), and 10*^-^*^13 ^l (black). The realizations show how the discrete nature of reactions plays an important role in the smallest volume and how the strength of noise gets smaller when the system volume increases. The effects of noise are however still detectable also in the largest volume. By taking a look at the PLC and CaPLCcomplex noise processes in (Figure 5A and 5B), it is easy to conclude that they are somewhat different. Therefore it is interesting to see how their behavior differs in the frequency domain. We estimated PSDs of the noise in the PLC and CaPLCcomplex concentrations using 10 second realizations of these processes. The frequency content of these noise processes as a function of volume can be seen in (Figure 5C and 5D). The noise in both species seems to have most of its power on the lower frequencies. Similar behavior was also observed in other species in the network. The PSDs for PLC and CaPLCcomplex seem to have different shapes (see Figure 5C and 5D). The noise in PLC realizations clearly has the dominating power on very low frequencies whereas the frequency content of noise in CaPLCcomplex realizations is distributed more uniformly on low frequencies. An interesting observation also is that the shapes of PSDs for species PLC and CaPLCcomplex seem to be same in all studied volumes. This can be seen clearly in Figure 6, where four PSDs in different volumes for PLC and CaPLCcomplex are plotted using the log-log-scale.

When low frequency noise is observed, it usually raises specific questions about the quality of noise. The shape of the PSDs in Figure 6A is similar to the kind of behavior that is typical for power law or 1/*f^α^*-noise processes. When these processes are studied in the frequency domain, the shape of the spectrum is determined by the power law *f^-α^*, where *f *is the frequency. 1/*f^α^*-noise processes have been found in several physical systems and they have been extensively studied [39]. A special case of these noise processes is the so-called 1*/f*-noise which cannot be characterized in the time domain. This makes 1/*f*-noise extremely hard to model and identify. In our simulations, the Gillespie SSA which practically simulates a Markov model produces a behavior for the noise in PLC concentration that clearly resembles some kind of 1*/f^α^*-noise. Although a Markov model is not capable of producing pure 1/*f*-noise, it is still of interest to study the quality of the noise processes in the context of power law noises. The slopes of the log-log-scaled spectra in Figure 6A are in the range [-1.8, -1.7]. A pure 1/*f*-noise process would have a slope of -1. This means that the behavior of the behavior of noise in PLC concentration is closer to the behavior of a random walk process (slope of -2) than 1/*f*-noise. It is still unclear if there exists 1/*f*-noise in real biochemical systems. Computational models and methods will play a crucial role in studying this issue in the future.

We conclude that the frequency domain analysis is a workable approach when studying noise in biochemical reaction networks. We showed that even the simple methodology that we used in this study can be successfully applied to assess these features. We propose that the frequency domain analysis for noise should always be performed when BRNs are modeled using stochastic approaches.

## Discussion

### Biochemical noise and computational techniques

In this study, we investigated noise processes occurring in a large biochemical network. The analysis was carried out in both the time and frequency domain. The numerical frequency domain analysis of this kind has been applied also in other simulation studies where periodic or quasi-periodic oscillations obtained from Gillespie SSA simulations have been of interest (see e.g. [25,40,41]). In this study, however, we concentrated on the quality of noise in pure noise processes instead of oscillations. Our results (on time and frequency domain) were in agreement with previous studies: the high-frequency noise is attenuated by the system structure [2], in small volumes discrete reaction events become more important [19], and when the volume is increased, the importance of noise slowly diminishes but does not disappear [27]. In addition to the previously presented results, we showed how the frequency content of a biochemical noise process changes as a function of volume. To our best knowledge, this kind of analysis has not been done before and we believe that our approach could be applicable in other studies as well. Especially it might shed light on the question of the quality of noise in different kinds of modeling approaches and it could be applied for example when benchmarking new approximate simulation approaches. To make the computational techniques used in this study easily accessible, we introduced a straightforward sampling algorithm for Gillespie SSA simulation results (see Figure 1). It is also obvious that the methods presented in this study are easy to implement and use. Therefore, the kind of analysis we present here could be carried out for example as a starting point for a more advanced frequency domain analysis.

Although our emphasis was on the frequency domain analysis, the time domain results of our study were of interest as well. We noted that the noise processes simulated using the stochastic Gillespie SSA do not deviate far from the ODE response. This kind of behavior shows the robustness of the network: although the environment and reaction events are noisy, the network still performs the same task. In addition, the coefficient of variation was used to describe the dependency between the strength of noise and volume in the time domain. Although the most of the simulated noise processes behave rather well, the large network also includes noise processes representing more unpredictable and irregular behavior.

Besides the methodological aspects of this study, the parallel computing proved to be an indispensable technique when massive BRNs simulations were performed. Without parallel computing, the simulation of 300 different volumes would have been impossible and we would have had to content ourselves with less inclusive results. Although the implementation of parallelism takes time, the benefits are so notable that the parallelization is unquestionably worth doing. We believe that the application of parallel computing will increase explosively in the field of computational systems biology and its subfields in the near future.

### Insights and future work

The results presented in this article give new insight to the quality of noise in one signal transduction network. In addition, the methodology can in principle be applied to the characterization of noise processes in any other similar system. The methods presented in this paper are widely applicable because almost all biological processes inherently represent some kind of variability. Without a proper analysis it is impossible to know if noise has any practical meaning. When we are dealing with data produced by computer simulations, we are able to fully control the whole process of data production. In order to extract all possible information from the results, new methodology should be developed and applied. Frequency domain analysis is widely applied in science in general and, as we have shown, it can, with minor modifications, be applied also to the analysis of Gillespie SSA simulation results. Frequency domain analysis is, however, just one of the numerous ways of analyzing stochastic simulation results from a new perspective. Basically all kind of methods that can be used to extract information from time-series data are potential tools in this particular area and we hope that this area of research will attain more attention. There still exist numerous challenges in the analysis of noise in BRNs. Our future work includes, for example, the testing and development of new analysis methods for examination of noise in subcellular systems. We are especially interested in noise processes representing 1/f-noise which we also discussed in this study. Our further interests include, for example, new modeling approaches such as non-Markovian models including delays and their capability of producing biologically realistic noise processes.

## Conclusions

In this study, we discussed how noise arising from molecular interactions in biochemical reaction networks can be examined using simulations and numerical frequency domain analysis. Biochemical reaction networks form the basic information processing mechanisms in biological systems and, in order to understand these mechanisms, we have to understand the stochastic phenomena affecting molecular dynamics. Stochastic modeling is an invaluable tool in this endeavor. We implemented a stochastic model for a large, realistic biochemical reaction network, performed massive parallel simulations, and analyzed the simulation results both in the time and frequency domain. We concentrated on the characterization of intrinsic noise appearing in a specific network. The simulation results showed that there are several kinds of noise processes in the network, all of them representing different kind of low-frequency fluctuations. The frequency domain behavior of biochemical noise processes was presented as a function of an altering system volume. The low-frequency nature of the noise processes in all studied volumes could be deduced from the estimated power spectral densities.

## Competing interests

The authors declare that they have no competing interests.

## Authors' contributions

JI, TM, MLL, and KR designed the study. TM collected the network model from literature. TM and JI implemented the stochastic and deterministic simulation procedures. JI implemented the algorithms needed in parallel simulations and in the analysis of results, performed simulations, analyzed the results, and wrote the manuscript. MLL and KR supervised the study. All authors read and approved the final manuscript.
